# Assessing and Promoting Cardiovascular Health for Adolescent Women: User-Centered Design Approach

**DOI:** 10.2196/42051

**Published:** 2022-12-19

**Authors:** Kolbi Bradley, Santiago J Arconada Alvarez, Amanda K Gilmore, Morgan Greenleaf, Aayahna Herbert, Melissa J Kottke, Maren Parsell, Sierra Patterson, Tymirra Smith, Mercedes Sotos-Prieto, Elizabeth Zeichner, Holly C Gooding

**Affiliations:** 1 Department of Pediatrics Emory University School of Medicine Atlanta, GA United States; 2 Georgia Clinical and Translational Science Alliance Atlanta, GA United States; 3 Emory University School of Medicine Atlanta, GA United States; 4 Department of Health Policy and Behavioral Sciences School of Public Health Georgia State University Atlanta, GA United States; 5 National Center for Sexual Violence Prevention, Mark Chaffin Center for Healthy Development School of Public Health Georgia State University Atlanta, GA United States; 6 College of Computing School of Interactive Computing Georgia Tech Atlanta, GA United States; 7 Jane Fonda Center Department of Gynecology and Obstetrics Emory University School of Medicine Atlanta, GA United States; 8 Emory Healthcare Atlanta, GA United States; 9 Department of Epidemiology University of North Carolina at Chapel Hill Chapel Hill, NC United States; 10 College of Design School of Industrial Design Georgia Tech Atlanta, GA United States; 11 Department of Preventive Medicine and Public Health School of Medicine Universidad Autónoma de Madrid Madrid Spain; 12 Centro de Investigacion Biomedica en Red Epidemiologica y Salud Publica Madrid Spain; 13 Department of Environmental Health Harvard T.H. Chan School of Public Health Boston, MA United States; 14 Children's Healthcare of Atlanta Atlanta, GA United States

**Keywords:** adolescent, heart disease, mHealth, digital health intervention, user-centered design, cardiovascular disease, CVD, women's health, risk assessment, young adults, assessment tool

## Abstract

**Background:**

Cardiovascular disease (CVD) is the leading cause of death among women in the United States. A considerable number of young women already have risk factors for CVD. Awareness of CVD and its risk factors is critical to preventing CVD, yet younger women are less aware of CVD prevalence, its risk factors, and preventative behaviors compared to older women.

**Objective:**

The purpose of this study is to assess CVD awareness among adolescent and young adult women and develop a lifestyle-based cardiovascular risk assessment tool for the promotion of CVD awareness among this population.

**Methods:**

This study used a 3-phase iterative design process with young women and health care practitioners from primary care and reproductive care clinics in Atlanta, Georgia. In phase 1, we administered a modified version of the American Heart Association Women’s Health Survey to young women, aged 15-24 years (n=67), to assess their general CVD awareness. In phase 2, we interviewed young women, aged 13-21 years (n=10), and their health care practitioners (n=10), to solicit suggestions for adapting the Healthy Heart Score, an existing adult cardiovascular risk assessment tool, for use with this age group. We also aimed to learn more about the barriers and challenges to health behavior change within this population and the clinical practices that serve them. In phase 3, we used the findings from the first 2 phases to create a prototype of a new online cardiovascular risk assessment tool designed specifically for young women. We then used an iterative user-centered design process to collect feedback from approximately 105 young women, aged 13-21 years, as we adapted the tool.

**Results:**

Only 10.5% (7/67) of the young women surveyed correctly identified CVD as the leading cause of death among women in the United States. Few respondents reported having discussed their personal risk (4/67, 6%) or family history of CVD (8/67, 11.9%) with a health care provider. During the interviews, young women reported better CVD awareness and knowledge after completing the adult risk assessment tool and suggested making the tool more teen-friendly by incorporating relevant foods and activity options. Health care practitioners emphasized shortening the assessment for easier use within practice and discussed other barriers adolescents may face in adopting heart-healthy behaviors. The result of the iterative design process was a youth-friendly prototype of a cardiovascular risk assessment tool.

**Conclusions:**

Adolescent and young adult women demonstrate low awareness of CVD. This study illustrates the potential value of a cardiovascular risk assessment tool adapted for use with young women and showcases the importance of user-centered design when creating digital health interventions.

## Introduction

Cardiovascular disease (CVD) remains the leading cause of death for women in the United States, despite decades of progress in risk factor detection and treatment [1,2]. Awareness of CVD and its risk factors is critical to reversing this trend, yet national data from the American Heart Association (AHA) Women’s Health Survey reveals that younger women aged 25-34 years are much less likely than older women to be aware of CVD and its risk factors, a trend that is worsening over time [3]. A previous study of young women aged 18-39 years participating in the National Health and Nutrition Examination Survey in 2011-2014 found a considerable number already had hypercholesterolemia, hypertension, and diabetes, yet many were unaware of their diagnosis [4]. Compounding this lack of CVD awareness, people of all ages tend to underestimate the likelihood of experiencing negative events and overestimate the likelihood of experiencing positive events [5]. Adolescents especially have been characterized as “young invincibles” with little consideration for their long-term health [6]. However, adolescents are cognitively capable of understanding short-, medium-, and long-term risks for CVD [7] and have been shown in prior studies to report motivation to act now to prevent future CVD [8].

Previous interventions to improve CVD awareness and mitigate CVD risk in adolescent and young adult (AYA) women have been limited to distinct populations, such as college students [9], or have focused on addressing only a few CVD risk factors at a time [10-13]. Mobile health (mHealth) interventions, defined as “medical and public health practice supported by mobile devices, such as mobile phones, patient monitoring devices, personal digital assistants, and other wireless devices” [14], are increasingly used to engage patients in CVD health promotion and behavior change [15-17]. There is a need to further explore the use of mHealth tools in diverse populations [18], including adolescents and women, and to optimize their use within clinical practice [17].

The Healthy Heart Score (HHS) is an existing online assessment tool that estimates one’s risk for CVD based on self-reported modifiable health behaviors [19]. The HHS was developed using epidemiologic data from adults of 40-75 years participating in the Nurses' Health Study and Health Professionals Follow-Up Study, who were then followed for over 24 years [19]. An algorithm consisting of age, smoking status, physical activity, diet, alcohol intake, and body mass index was found to adequately predict their 20-year risk of a CVD event. We subsequently validated the use of this same algorithm in Black and White young adults aged 18-30 years participating in the Coronary Artery Risk Development in Young Adults cohort study and found that it predicted risk of a CVD event before 55 years of age as well [20]. The HHS is especially well suited for use with younger women because it does not require clinical measurements or laboratory assessments, assesses modifiable health behaviors, and performs best in those without established CVD risk factors [21].

In this project, we aimed to create a developmentally appropriate mobile CVD risk assessment tool based on the HHS algorithm for use in wellness, mental health, and reproductive health visits for young women in a diverse community in Atlanta, Georgia. In the first phase of the study, we assessed the baseline understanding of CVD and its risk factors in our participant population using the AHA Women’s Health Survey [3] to target the new tool at existing knowledge gaps. In the second phase, we presented the existing adult HHS online risk assessment to young women and their health care providers (HCPs) and solicited their ideas for necessary adaptations to make the tool more teen-friendly. In the third phase, we used an iterative user-centered design process to continuously adapt and test revisions to the tool. The final product is an online tool developed with and for young women to address identified gaps in their CVD awareness and promote positive behavior change.

## Methods

### Study Population

AYA women, aged 13-24 years, were recruited from a primary care practice and an adolescent reproductive health clinic in southeast Atlanta for all 3 phases of the study. The adolescent reproductive health clinic was selected for recruitment in addition to the primary care clinic because many young women are only present for reproductive health care [22], and in our prior work, young women suggested tying cardiovascular health promotion to their reproductive health concerns as a viable prevention strategy [23]. Southeast Atlanta is a diverse location of Atlanta, with over 70% of the population identifying as Black or African American [24]. The majority of households, served by these 2 practices, report under US $50,000 per year in annual income [24]. The primary care practice provides primary care, mental health care, and sexual health care services to over 1700 adolescents aged 13-21 years annually and is staffed by adolescent medicine physicians, nurse practitioners, a psychotherapist, and a supporting clinical team. The reproductive health clinic provides confidential sexual health services to young women aged 13-24 years and is staffed by gynecologists, nurse midwives, a health educator, and a supporting clinical team.

### Ethical Considerations

This study was approved by the Emory University Institutional Review Board, the Children’s Healthcare of Atlanta Institutional Review Board, and the Grady Health System Research Oversight Committee (approval number 00001418). Informed consent was obtained from all surveyed and interviewed participants and their guardians, except in cases where minors presented alone to the clinic, in which an approved waiver of guardian consent was invoked. All data collected from surveys and interviews were deidentified and stored on Emory University’s password-protected servers. A US $25 gift card was given to those who participated in surveys and recorded interviews. A waiver of written informed consent was approved by the aforementioned review boards in cases of observational feedback. No identifying information was recorded for observational feedback, and no financial compensation was provided.

### Study Procedures

#### Phase 1: Baseline Assessment of Adolescent Understanding of CVD Risk

Young women presenting for annual well visits from March 2021 to December 2021 were eligible for this phase of the study. Trained research coordinators approached young women in the waiting room and asked them to complete a modified version of the AHA Women’s Health Study survey [25]. Participants provided electronic informed consent before agreeing to complete the survey. A total of 67 young women completed the survey. We calculated and reported descriptive statistics using SAS 9.4 (SAS Institute).

#### Phase 2: Adolescent and HCP Feedback on the Existing CVD Screening Tool (HHS)

Young women and HCPs from both practices were eligible for this phase of the study, which occurred from August 2021 to December 2021. All participants completed the existing HHS CVD risk assessment online and answered questions administered by a member of the research team, including feedback on the HHS architecture (layout, word choice, use of visuals), conditions under which they thought adolescents would engage with the assessment (eg, at school or the doctor’s office), and general suggestions for improvement. Four initial interviews occurred over 1 hour using Health Insurance Portability and Accountability Act-compliant Zoom during the COVID-19 pandemic. Due to difficulty scheduling the Zoom interviews, we switched to a 15-minute semistructured interview in the clinics in October 2021, addressing the same topics in an abbreviated form. While interviews were conducted in quiet areas, we allowed friends, family members, or guardians to be present during interviews if preferred by the participant. We compared the results from the in-person interviews to those from the Zoom interviews to check for differences; no qualitative differences were found in the results. A total of 11 adolescents and 10 HCPs completed interviews. All interviews were recorded and audio-transcribed verbatim. Due to the failure to record, 1 interview was lost, leaving 10 AYA interviews for textual analysis. All participants in this phase provided written informed consent to be interviewed and recorded. The HCP interview guide is available in the Multimedia Appendix 1 and the AYA interview guide is available in the Multimedia Appendix 2.

Transcriptions were read by the research team members, including an adolescent medicine physician, a clinical research coordinator, and a medical student. Using an inductive coding approach, the research team met to discuss themes and decide on a set of codes and subcodes (see Table 1). Transcriptions were then coded in teams of 2 using Dedoose, version 8.3.10 (SocioCultural Research Consultants). After double-coding the first 3 transcripts, the team met to review the code application and ensure agreement. Any disagreements were discussed during the team meeting. This process was repeated to ensure consistency in coder agreement.

#### Phase 3: Adolescent CVD Screening Tool Development

**Figure 1:**
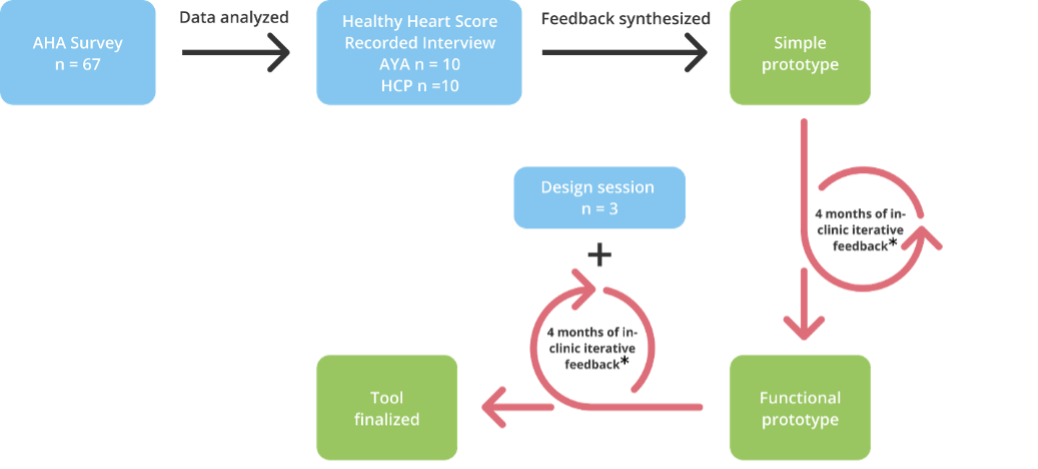
Study flow chart. AHA: American Heart Association; AYA: adolescent and young adult; HCP: health care provider. *A nutritional epidemiologist and clinical psychologist were consulted throughout phase 3’s iterative design process to ensure the tool was aligned with principles of motivational interviewing, accurate serving size depictions, and several health psychology frameworks.

The clinical research team collaborated with the programming and design team to synthesize findings from the AHA survey from phase 1 and HHS interviews from phase 2 into a simple wireframe prototype of a new CVD risk assessment tool for phase 3. A member of the clinical research team then presented the prototype to young women in the waiting rooms of the 2 practices from October 2021 to June 2022. Patients were asked to provide brief feedback on various topics, including layout, content, usability, and areas for improvement. The research team member took notes and presented this feedback to the programming and design teams to inform subsequent iterations of the tool. All data was observational and anonymous, and written informed consent was not required per the institutional review board approvals for this phase. During the fourth month of this user-centered design process, the team facilitated an hour-long design session with 3 participating adolescents to supplement in-clinic feedback using the Ten Design Heuristics [26]. The team consulted with a clinical psychologist and nutritional epidemiologist throughout this iterative design phase to ensure that adapted features and content appropriately used behavioral and health psychology frameworks such as motivational interviewing [27], the health belief model (HBM) [28], the behavioral intervention technology model [29], and social cognitive theory [30], as described further in the Results section. The complete iterative design process, including all 3 phases, is depicted in Figure 1.

## Results

### Phase 1: Baseline Assessment of Young Women’s Understanding of CVD Risk

Of the 67 participants who completed the survey, 47 (71.2%) were 15-17 years of age, 18 (27.3%) were 18-21 years of age, 1 was older than 21 years, and 1 did not state her age. Most respondents identified as Black (56/67, 75.4%) and 13 of 67 (22.4%) identified as Hispanic. The majority of the participants (48/67, 71.6%) reported that their primary caregiver had less than a college education. Only 10.5% (7/67) of the participants were aware that CVD is the leading cause of death among women in the United States. Participants were asked which health topics they discussed with their HCP. Very few reported that their HCP discussed their personal risk of CVD (4/67, 6%) or their family history of CVD (8/67, 11.9%) with them. Of the remaining topics surveyed, 46.3% (31/67) reported discussing their exercise habits; 49.3% (33/67) reported discussing their weight; and 26.9% (18/67) reported discussing none of the listed topics with their provider. Participants were also asked to identify major causes of CVD. Less than half of participants were aware that low physical activity (19/67, 28.4%), diabetes (27/67, 40.3%), smoking (29/67, 43.3%), and high cholesterol (33/67, 49.3%) contribute to CVD. High blood pressure (37/67, 55.3%) and being overweight (44/67, 65.7%) were identified as major causes by over half of respondents. Participants were also asked what CVD preventative behaviors they took within the past year. Fewer than half of the participants reported any of the CVD prevention behaviors surveyed, with only 4.5% (3/67) reporting maintaining healthy cholesterol, 10.4% (7/67) reporting maintaining a healthy blood pressure, 11.9% (8/67) reporting reducing sodium/salt intake; 23.9% (16/67) reporting reducing sugar intake; 38.9% (26/67) reporting attempting to lose weight, and 49.3% (33/67) reporting getting physical exercise.

### Phase 2: Adolescent Feedback on the Existing Adult CVD Screening Tool

A total of 10 young women aged 13-21 years and 10 HCPs aged 29-65 years contributed interviews for the analysis. All the adolescent participants identified themselves as Black. All HCPs identified as women, with 6 identifying as Black and 4 identifying as White. One HCP also identified as Hispanic. HCPs held various positions and degrees, including clinical assistant (n=1), nurse (n=3), mental health clinician (n=1), physician (n=2), advanced practice provider (n=2), and health educator (n=1).

Table 1 summarizes key themes and subthemes derived from participant interviews. Consistent with the survey data, adolescent participants demonstrated low knowledge of their own risk for CVD prior to completing the HHS, but felt they had increased CVD awareness after completing the HHS assessment. HCPs acknowledged that while they felt comfortable talking about CVD-related topics such as nutrition, having access to more resources would help increase their comfort discussing CVD with patients. Both adolescents and HCPs suggested: (1) adding more teen-friendly food and activity options, (2) using explanations and examples throughout the tool (including visuals), (3) improving the layout and design of the tool, (4) using motivating language more effectively, and (5) minding the reading level of the tool. Both adolescents and HCPs expressed concern about teens providing honest answers in the assessment as well as ongoing engagement with behavior change goals. The barriers and facilitators to use such a tool in practice were also shared, with HCPs speaking directly about limited clinic resources and time. Both HCPs and adolescents brought up the potential for fear of judgement, shame, and embarrassment surrounding answers to certain questions (eg, weight, alcohol intake, and nicotine use), as well as fear of learning about one’s CVD risk. After completing the assessment, many adolescents demonstrated both curiosity and shock at their predicted risk. HCPs commented on additional barriers (eg, healthy food affordability) and facilitators of adolescent health behavior change (eg, working with parents to assist in lifestyle changes). To facilitate the implementation of the assessment in practice, HCPs recommended shortening the assessment as time is often limited in the clinical setting. Both groups felt that going through the HHS assessment itself could be a cue to action for adolescents to change their behavior, consistent with the HBM [28].

**Table 1:** Coding themes and representative quotes from 10 adolescent and young adult women (AYA) and 10 health care providers (HCPs) giving feedback on the Healthy Heart Score risk assessment.

Themes	Subthemes	Illustrative quotes
Health knowledge	HCP CVD^a^ knowledge and comfortability Teen knowledge of heart attack and stroke Tool as increasing health awareness Sources of knowledge	“…people’s lack…of awareness of that risk or seriousness of that risk, too, especially for young women. They…may just feel like that’s not a thing they necessarily should be worried about...” HCP “Because I think if I eat healthy and take care of myself right, I don’t think that’s going to happen to me, but I don’t know” AYA “I guess things…that surprised me a little, like my instincts with certain foods like, that didn’t make me think, oh man, I’m going to get like a heart attack. But made me think OK, like maybe I should try a little more on that.” AYA
Content of risk assessment tool	Choices Explanations and examples Layout and design Length Wording Motivating language Reading or content level Teen-friendly Visuals	“I feel like, the reading, like on some of them, like it’s helpful, but it’s also a lot. Like I didn’t read through it.” AYA “Well, [the activity choices] were perfectly fine for my kinds of activities, but I can imagine for kids they need to be different because of organized sports.” HCP “I think the time estimates are maybe going to be hard for some people to make like you have to do a little bit of mental math.” HCP [On what was confusing] “The, like how might categorize [physical activity or food intake] like the one, two, three per week.” AYA “I guess it’d be you wanted to have a Spanish version” HCP
Conditions to tool usage	Honest engagement with tool Initial assessment Ongoing work Barriers to tool usage Facilitators to tool usage	“I know a few people my age who do drink. And so, I feel like they wouldn’t answer honestly.” AYA “It would be nice to be able to communicate with [teens that] are feeling the same way that actually want to improve their health […] you can relate to someone in the process.” AYA “Sometimes just due to scheduling and the timing of things, you may not have time enough to complete the survey” HCP “This tool is easy, and with technology now, patients are tired of paper.” – HCP
Emotional reaction	Curiosity about personal results Fear of judgment Fear of results Shame or embarrassment Sharing personal details Surprise or shock Weight	“I’m scared […] because it’ll probably be like, oh well, you probably need to get better with your health or [you’ll have] a heart attack.” AYA “It was kind of shocking to see the result.” AYA “Especially girls. Like they’d be real self-conscious about [their weight]. And so, with the way society and stuff is, and all the pressure to look a certain way and be a certain size or weight this much.” AYA “It’s just a form of education or awareness…I don’t want to have our teens freaking out once they see that.” HCP
Health behaviors	Barriers to behavior change Facilitators to behavior change Existing teen behavior	“High fiber cold cereal? I really don’t eat cereal like that. I don’t really eat breakfast…” AYA “Affordability is an issue as far as what’s at home, what’s available at home or if the teen is even cooking, or does mom cook every day.” – HCP
Motivation	Tool as cue to action External motivation Internal motivation	“I definitely need to get serious about my weight…and move my body more.” AYA “I have a lot of people in my family that have diabetes, high blood pressure, and high blood sugar.” AYA
HCP role in CVD risk assessment	N/A^b^	“One of the focus points of the well visit is to assess people’s risk and to address that either by doing further screenings like checking their cholesterol or based on like the data that you have of what their risk might be kind of talking to them about lifestyle changes that could help prevent cardiovascular disease.” HCP

^a^CVD: cardiovascular disease.

^b^N/A: not applicable.

### Phase 3: Adolescent CVD Screening Tool Development

Using data from the phase 1 survey and phase 2 interviews, the programming and design team worked with the clinical research team to develop an initial simple prototype of the tool. In concordance with the behavioral intervention technology model [29], the research team further defined the aims of our tool, focusing on increasing physical activity and the consumption of fruits, vegetables, and grains, and decreasing the consumption of sugar, red meat, and tobacco. With the design team, behavioral strategies were identified, such as education, feedback, agency emphasis, and goal setting. This resulted in an initial prototype that addressed low-CVD prevention awareness activities identified in phase 1, including more relatable teen activities such as team sports (eg, cheerleading, basketball) and food options (eg, oatmeal, popcorn). It also depicted activities and foods with cartoon icons, as the interview findings suggested using visual representations to aid comprehension. Next, the team addressed HCP concerns about assessment length, reducing the tool’s length by combining individual foods (eg, cereal, soda, hot dogs, almonds, apples, green beans) into food groups (high-fiber grains, sugary or sweetened drinks, red meats, processed meats, nuts and seeds, fruits, and vegetables), reducing the total number of nutrition questions from 23 to 14. Similarly, we grouped all vigorous activities and moderate activities together into these 2 categories, reducing the total number of physical activity questions from 12 to 4. The total number of questions was reduced from 35 to 18 with these changes. To help avoid feelings of judgment surrounding body size, we deprioritized weight by moving this question to the end and removing feedback on body mass index. We also avoided using any leading or judgmental language in the feedback that may imply a “correct” answer and dissuade teens from answering honestly, opting instead to use more neutral language as suggested by motivational interviewing [27].

This initial storyboard prototype was then shown to teens in the clinic waiting rooms to further refine the examples and language in the questions. Using the first round of in-clinic feedback on the simple prototype, a functional web-based prototype was then created that fully showed risk, results, and recommendations. Due to the overall low 20-year CVD risk that adolescents have because of their age, the calculated relative scores were translated into point values and shown to the user. These points correspond to the relative importance of the individual elements in the original HHS algorithm. At the end of the assessment, feedback was given in the form of graphs that portrayed results compared to a healthy individual of the same age. TikTok videos demonstrating healthy eating and physical activity tips were initially included based on adolescent suggestions for engaging content. The TikToks were later removed due to the team’s inability to find high-quality, evidence-based videos on the platform, and replaced with nutrition and activity advice from the AHA combined with GIFs (moving visuals).

**Figure 2:**
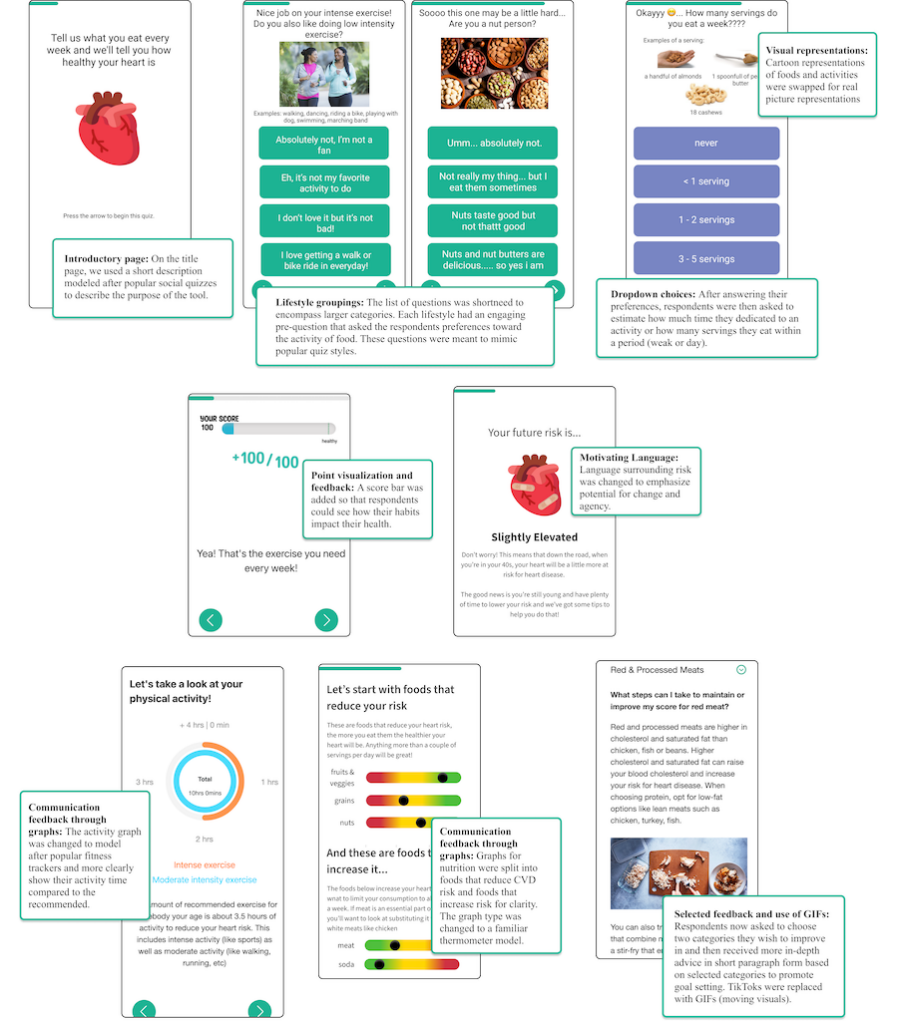
Final prototype adaptations and explanations.

This functional prototype was then shown to additional young women in the clinic waiting rooms and to 3 adolescents in the design session. Further feedback led to refinements, including clearly highlighting the purpose of the tool in bold and addressing both visual inconsistencies (eg, the dropdown for choices sometimes blocked the questions) and semantic issues (eg, some questions did not have the option to select “never”). Points accrued with each health behavior reported were highlighted to improve engagement throughout the assessment. Incorporating motivational interviewing techniques [27], we attempted to emphasize adolescents’ autonomy by giving respondents options on which areas they would like to receive additional feedback for. We used the foundations of the HBM [28] as guidance for the feedback, focusing first on raising awareness of an individual’s CVD susceptibility and then linking this to specific actions to reduce their CVD risk, along with addressing barriers with specific tips. Language within the feedback also focused on promoting adolescents’ self-efficacy and agency to change their scores. Multiple iterative changes were made to the feedback graphs to improve the communication of results. A representation of the final tool can be seen in Figure 2.

## Discussion

### Principal Results

CVD awareness, knowledge of CVD risk factors, and preventive behaviors were remarkably low among the young women surveyed in this population, which is consistent with other national [3] and regional [25] studies. Few young women reported having never spoken to an HCP about their personal risk for or family history of CVD, despite this study occurring in a region of the United States with some of the highest morbidity and mortality from CVD [31]. Together, these findings confirmed the urgent need to create a tool to promote CVD awareness, teach preventative measures, and prompt discussion of CVD between young women and their HCPs.

We chose the existing adult HHS as our initial tool for adaptation based on its alignment with the knowledge gaps identified in the survey and existing evidence for its ability to predict early CVD events in both middle-aged and young adults [19,20]. We found that such a tool can indeed prompt adolescents to reflect on their health and habits, as has been shown in a study with middle-aged adults [32]. Similarly, HCPs discussed the low CVD awareness amongst their young female patients and agreed this risk assessment tool could be a value-added resource to improve awareness. Further discussion with both young women and HCPs revealed the need for such a tool to incorporate teen-friendly foods and activities, clear and concise language, and pictures to improve overall understanding and the accuracy of responses. One issue presented by both HCPs and AYAs was the concern that AYAs would not be honest when answering the survey due to certain topics such as weight, alcohol intake, and nicotine use. It should be noted that the presence of friends, family members, or guardians during interviews may have also affected the candor of participants and therefore the information collected. Consideration of when and where the tool is administered should be taken to ensure that AYA respondents feel they can answer the questions honestly. Additionally, HCPs presented potential clinical barriers, to implementation, including time, and a lack of their own CVD knowledge. Other studies have discussed similar clinical barriers such as practice buy-in, obtaining cooperation and resources, and lack of HCP subject knowledge that have impacted successful intervention implementation [33,34]. Mitigating these challenges is essential to successfully integrating a cardiovascular risk assessment tool into a clinical practice and will be the focus of future studies.

Our iterative design process built upon this feedback and continuously incorporated input from young women through brief interviews in clinic and a design session, resulting in an age-appropriate and culturally tailored cardiovascular risk assessment tool. The input gathered allowed us to make changes to the tool to improve its aesthetic and enjoyability, along with its clarity and functionality. This process highlighted the importance of user-centered design when creating mHealth interventions. Researchers typically use a top-down approach when creating mHealth interventions, preferring to integrate evidence-based care directly into the digital sphere, resulting in mHealth interventions that may not effectively engage their audience [35]. Research has shown that while adolescents are one of the biggest consumers of mHealth interventions, many such tools do not effectively engage them [35]. Studies that have used a user-centered design process have shown great promise in engaging their target population [15]. Involving adolescents in the design process through workshops and direct interviews can help create tools with features that will engage this population. Combining this user-centered design with evidence-based protocols allows for more effective interventions that not only provide evidence-based guidance but also reach and engage users [36].

### Limitations

A major strength of this study is its mixed-methods approach, which leads to a tool that is uniquely tailored to the needs of this specific patient population of young women but is also likely applicable to young women from other backgrounds. Our patient population consisted of young women from minorities and low-income backgrounds, a population whose preferences and experiences are noted as lacking within mHealth intervention development but essential to creating broadly applicable tools [17,36]. The original tool from which our tool is adapted has also been shown to accurately predict the risk of early CVD events in young adults [20]. However, there are limitations to our work. A convenience sample was used to collect survey data and input on the new tool development. As a result, our findings may lack generalizability beyond our local sample and will need to be tested in other populations of adolescents. We also made several adaptations to the validated cardiovascular risk prediction tool to make it usable and acceptable for teens, such as consolidating questions, modifying phrasing, and adjusting the calculation to predict relative risk, and thus, the tool may no longer represent the actual numerical risk of an early CVD event with the same predictive accuracy. Finally, whether the tool prompts true behavior change and correlates with CVD risk reduction will be explored in future studies.

### Conclusions

The young women in this study and others consistently demonstrate low awareness of CVD and CVD prevention. More resources are needed to educate young women about CVD and promote CVD discussions with HCPs. An online risk assessment tool is one potential resource that can help increase awareness of CVD if tailored to the needs and preferences of this population. Further research is needed surrounding the feasibility and usability of such a tool in clinical practice, as well as its efficacy in raising CVD awareness and inspiring young women to take action for their future heart health.

## Appendix

Multimedia Appendix 1 Health care provider semistructured interview guide.

Multimedia Appendix 2 Adolescent semistructured interview guide.

## Abbreviations

AHA: American Heart Association
AYA: adolescent and young adult
CVD: cardiovascular disease
HBM: health belief model
HCP: health care provider
HHS: Healthy Heart Score
mHealth: mobile health
