# Supplementary material for: Assessing and Promoting Cardiovascular Health for Adolescent Women: User-Centered Design Approach
Source: JMIR Form Res. 2022 Dec 19;6(12):e42051. doi: 10.2196/42051 (PMC9808721; doi:10.2196/42051)
Supplement: Multimedia Appendix 1 [file formative_v6i12e42051_app1.pdf]

<u>Semi-Structured Interview for Health Care Provider Participants (Aim 1)</u>
(C3.2.v)

Clinical Research Coordinator Script: Thank you for participating in this interview about integrating cardiovascular health prevention into your clinical practice. Participation in this study is completely voluntary and you may decline to answer any of the questions. There are no right or wrong answers – I only want to know your opinion. The interview should take less than 30 minutes to complete, and all information we collect will be completely anonymous. You will be assigned a number at random and your data will be recorded by this number, not by your name. Responses cannot be linked back to you and sensitive responses cannot be acted upon.

<u>General Questions</u>
Describe your role in the prevention of cardiovascular diseases.

How comfortable are you in assessing the CVD risk of your young women patients?

What are the barriers to providing CVD prevention care in your practice? What are the facilitators?

<u>Feedback on the Healthy Heart Score Tool and Sample Diabetes Prevention Program Module</u>
After completing the Healthy Heart Score, what is your initial opinion of the Health Heart Score as a cardiovascular disease risk prediction tool?

What is your opinion about the length of the assessment for patient use?

Where would be the best place to utilize this assessment? (i.e. in waiting room, in examination room, as a follow up at home)

What are your thoughts on the food frequency questions?

What are your thoughts on the physical activity related questions?

What would be the most effective way to offer this assessment? (i.e. patients take assessment directly, health care provider reads assessment to patient)

What is your opinion of the sample handout and video?

What is your opinion of the health literacy level of the Healthy Heart Score assessment? Of the handout? Of the video?

If utilized correctly, do you think that an online tool consisting of the risk assessment and handouts/videos could be a valuable part of CVD risk education?

Do you feel that such a tool can be used in your practice to educate your patients in healthy lifestyles to prevent the development of clinical risk factors? Why or why not?

Can you foresee any barriers against using this tool in your practice?
