# Supplementary material for: Assessing and Promoting Cardiovascular Health for Adolescent Women: User-Centered Design Approach
Source: JMIR Form Res. 2022 Dec 19;6(12):e42051. doi: 10.2196/42051 (PMC9808721; doi:10.2196/42051)
Supplement: Multimedia Appendix 2 [file formative_v6i12e42051_app2.pdf]

## Semi-Structured Interview for Adolescent and Young Adult Participants (Aim 1)

*Clinical Research Coordinator Script: Thank you for participating in this interview about young women's heart health. We are taking a tool that was made many years ago for older adults that has been useful, and we would like to adapt it to teens like you. So we need to know all of the parts of the tool that you think are not helpful, don't apply to you, or that you don't understand. Some of the things might even seem offensive, like telling you what to eat or about your weight, so please let us know what you don't like about this so we can adapt it to teens and make it useful for teens like you. Participation in this study is completely voluntary and you may decline to answer any of the questions. There are no right or wrong answers – I only want to know your opinion. The interview should take less than 45 minutes to complete, and all information we collect will be completely anonymous. You will be assigned a number at random and your data will be recorded by this number, not by your name. Responses cannot be linked back to you and sensitive responses cannot be acted upon. We recommend that you speak with doctor at the CHOA Adolescent Clinic if the content of this interview concerns you.*

### Overall Questions

Do you know what a heart attack is or what it means? What about a stroke?

Describe what you know about preventing things like heart attacks and strokes, or brain attacks.

How likely do you think you are to have a heart attack or stroke in the next 10 years? Why did you choose this answer?

How likely do you think you are to have a heart attack or stroke in your lifetime? Why did you choose this answer?

### Healthy Heart Score – Section 1

Looking at this page, what do you think this is for?

Did any of the questions trick you?

What did you think of the gender question? Do you think it should include more than just male/female?

### Healthy Heart Score – Section 2

How did you feel answering these questions?

Did you feel like you could answer the questions accurately?

Is there anything that may help you figure things out a little better or easier?

What are your thoughts on the physical activity related questions? What would make them easier to understand? Are there any activities missing? Are there any activities that aren't relevant to you?

### Healthy Heart Score – Section 3

Were any of these questions confusing?

Are the answer choices confusing in any way? Should we include more or less choices?

Are there things that could help you answer the questions? What about pictures or animations?

**Healthy Heart Score – Section 4**

Were the > or < signs confusing? Should we change this in any way?

Did you know what things like high/low fiber cold cereal bran, or added germ were? How could we make these things better to understand? More examples, pictures, animations?

If someone like myself or your doctor wasn't sitting here, would you keep going through the survey?

Would you like to see a visual to estimate your servings or was the dropdown menu good enough for you?

Are these questions interesting to you?

**Healthy Heart Score – Section 5**

What did you think of the examples for processed meat?

Is there anything you didn't understand?

What are your thoughts on the food frequency questions? What would make them easier to understand? Are there any foods missing? Are there any foods that aren't relevant to you?

**Healthy Heart Score – Section 6**

How did it make you feel when you read the first question about alcohol? Even if it was for teens, how would answering that question make you feel? Does it stress you out?

What about the smoking question?

What do you think is at the end of this? What do you expect that to look like or what would you imagine it to look like?

**Healthy Heart Score – Summary**

How do you interpret what you see on the page?

Where did your eyes go first?

What is the big message you're getting form results? How does that make you feel?

Do you feel like you need to change anything?

**Healthy Heart Score – Lifestyle Recommendations**

Where do your eyes go first?

How are you interpreting this?

Can you summarize what the right sections are saying? What if none of that was there?

**<u>Healthy Heart Score – Diet Recommendations</u>**

Where do your eyes go first?

What is initial reaction?

How does it make you feel? Why?

Is it stressing you out?

Does it make you feel good/bad/scared? Do you feel overwhelmed?

How does seeing 'more' make you feel? Where do you think you should start?

What is the main takeaway? What is this trying to tell you?

If you were to walk away right now, how would you feel about your health?

Would you take the time to read all of this or do the red/green/yellow colors give you enough information?

Do you feel like you've received any conflicting information?

**<u>Feedback on the Healthy Heart Score</u>**

Did the Healthy Heart Score tool change your opinion about your likelihood of having a heart attack or stroke? If yes, in what way? If no, why not?

After completing the Healthy Heart Score, do you think it is a good way to help prevent heart problems? Why or why not?

What did you think about the length of the survey? Was it too long or too short?

Where would be the best place to take this survey? (i.e., in waiting room, in examination room, as a follow up at home)

Would you feel more comfortable taking the survey yourself, or having a health care provider guide you through the survey/ask you the questions? Why?

How would you rate your understanding of the questions being asked in this survey if 1 is little understanding and 5 is full understanding?

**<u>Feedback on Behavioral Interventions</u>**

*Clinical Research Coordinator Script: Now I am going to ask you about some things that we may be able to combine with the Healthy Heart Score tool to help teens improve their heart health. Again, the goal here is for you to tell us everything you don't like about this so we can improve it for teens like you. It would also be helpful to know what you like, and what you think*

*is missing that teens need to know. Remember, this was made for older adults so we need to know what should be changed for teens.*

Lots of things motivate young women to make choices about their health. What are some things that really motivate you to make choices about your health?

If we made suggestions or provided coaching/texting/etc to help teens improve their diet/physical activity based on their results from the survey, do you think we should speak to these motivations you mentioned – if so, which ones?

Do you think a health coach or someone who can guide you about how to use this information would be helpful?

How would you like to receive this information – as a text message? As a direct message as part of an app you download on to your phone? As part of a private social media group? Any other ideas?

How often would you like to receive this information?

Would you like to communicate with other teens who are getting these same messages? If so, how? Via social media, via group text, via a website, or some other way?

How likely are you to use tools like this after you have left the clinic?

What should we call this application?
